# VEGF Signaling and Targeted Therapies in Cancer: Molecular Mechanisms, Clinical Applications, Resistance, and Cardiorenal Toxicities

**DOI:** 10.3390/ijms27209376

**Published:** 2026-10-21

**Authors:** Abhishek Mishra, Md. Abdul Hye Khan, Ripa Jamal, Ilham Kadhim, John D. Imig

**Affiliations:** 1 Department of Pharmaceutical Sciences, College of Pharmacy, University of Arkansas for Medical Sciences, Little Rock, AR 72203, USA; ikadhim@uams.edu (I.K.); jimig@uams.edu (J.D.I.); 2 Arkansas Children’s Hospital, Little Rock, AR 72202, USA; ripajamal13@gmail.com

**Keywords:** vascular endothelial growth factor (VEGF), tumor angiogenesis, VEGF receptor tyrosine kinase inhibitors, VEGFR signaling, cardiovascular and renal toxicity, epoxyeicosatrienoic acids (EETs)

## Abstract

Vascular endothelial growth factor (VEGF) signaling is a central regulator of tumor angiogenesis and a major therapeutic target in oncology. VEGF-neutralizing agents and VEGF receptor tyrosine kinase inhibitors (VEGFR-TKIs) confer clinical benefit in multiple solid malignancies, but resistance and cardiovascular and renal toxicities can limit durable treatment. This review summarizes VEGF ligands, VEGF receptors, and the principal signaling mechanisms relevant to angiogenesis and lymphangiogenesis, with emphasis on VEGFR2 and VEGFR3 biology. We then examine established VEGF-targeted therapies, representative clinical evidence for VEGFR-TKIs, mechanisms of therapeutic resistance, and VEGF inhibitor-associated hypertension, endothelial dysfunction, proteinuria, and kidney injury. Particular attention is given to strategies for improving the therapeutic index of VEGF pathway inhibition, including risk assessment and toxicity monitoring, immunotherapy combinations, and emerging mechanism-based approaches. Modulation of the epoxyeicosatrienoic acid (EET)/soluble epoxide hydrolase axis is discussed as a promising experimental strategy for cardiorenal protection; however, current support is predominantly preclinical and requires clinical validation. Integrating tumor vascular biology with mechanisms of normal vascular and renal homeostasis may facilitate safer and more effective VEGF-directed cancer therapy.

## 1. Introduction

Angiogenesis, the formation of new blood vessels from pre-existing vasculature, is a tightly regulated physiological process essential for embryonic development, tissue growth, wound healing, and reproductive function [1,2]. Under normal conditions, angiogenesis is controlled by a balance between proangiogenic and antiangiogenic factors that maintain vascular homeostasis. Disruption of this balance contributes to numerous pathological conditions characterized by either insufficient vascularization or excessive vessel growth, including ischemic diseases, chronic inflammation, and cancer [2,3]. Consequently, angiogenesis has become a major focus of biomedical research and therapeutic intervention.

Given the central role of angiogenesis in vascular biology, considerable effort has been devoted to identifying its key molecular regulators. Among these, vascular endothelial growth factor (VEGF) is recognized as one of the most potent and extensively studied proangiogenic signaling molecules [1,2,3]. Since its discovery as both a vascular permeability factor and endothelial cell-specific mitogen, VEGF has emerged as a master regulator of vascular development and remodeling [3,4]. The VEGF family comprises VEGF-A, VEGF-B, VEGF-C, VEGF-D, and placental growth factor (PlGF), which signal through the receptor tyrosine kinases VEGFR1, VEGFR2, and VEGFR3 [3,5]. Activation of these receptors regulates endothelial cell proliferation, migration, survival, differentiation, vascular permeability, and lymphangiogenesis through signaling pathways such as phosphoinositide 3-kinase/protein kinase B (PI3K/AKT), phospholipase Cγ/protein kinase C (PLCγ/PKC), and mitogen-activated protein kinase/extracellular signal-regulated kinase (MAPK/ERK) [3,5,6]. In addition to promoting angiogenesis, VEGF signaling plays a critical role in endothelial cell survival, nitric oxide production, vascular remodeling, and maintenance of vascular homeostasis [4,5,6].

While tightly controlled VEGF signaling is indispensable for normal vascular function, dysregulation of this pathway contributes to numerous diseases, including cancer, diabetic retinopathy, age-related macular degeneration, rheumatoid arthritis, psoriasis, and chronic inflammatory disorders [3,6,7]. Among these conditions, cancer represents the most extensively studied example of VEGF-driven pathology because tumor growth and metastasis depend on the establishment of a functional vascular network. As rapidly growing tumors outpace their blood supply, hypoxic conditions stabilize hypoxia-inducible factor-1α (HIF-1α), which stimulates VEGF expression and promotes pathological angiogenesis [6,7,8]. The resulting tumor vasculature is structurally and functionally abnormal, exhibiting increased permeability, poor organization, impaired vessel maturation, and heterogeneous blood flow. These abnormalities contribute to tumor hypoxia, metastatic dissemination, therapeutic resistance, and inefficient drug delivery [7,8,9].

Cancer imposes an enormous and growing global health burden. According to the Global Burden of Disease (GBD) 2019 analysis, there were approximately 23.6 million incident cancer cases and 10.0 million cancer deaths worldwide in 2019, with cancer accounting for approximately 250 million disability-adjusted life years (DALYs) [10]. From 2010 to 2019, the absolute numbers of incident cancer cases, cancer deaths, and cancer-related DALYs increased by 26.3%, 20.9%, and 16.0%, respectively [10]. Cancer ranked second only to cardiovascular diseases in global deaths, years of life lost, and DALYs, and approximately 96.9% of cancer-related DALYs were attributable to years of life lost, emphasizing the major contribution of premature mortality to the overall cancer burden [10].

More recent GBD 2021 estimates further demonstrate the persistence of this global burden. In 2021, cancer accounted for approximately 14.57% of all deaths and 8.8% of total DALYs worldwide, with approximately 9.89 million cancer-related deaths [11]. Furthermore, an estimated 100.30 million cancer-related DALYs were attributable to assessed risk factors, representing 39.59% of total cancer DALYs [11]. These findings demonstrate that, despite advances in cancer prevention, diagnosis, and treatment, malignancy continues to account for a substantial proportion of global premature mortality and health loss.

The burden of cancer also has major socioeconomic implications. Beyond mortality and disability, cancer generates substantial direct healthcare expenditures and indirect costs associated with disability and productivity loss. A systematic review and meta-analysis examining cancer-related costs in relation to DALYs estimated a pooled total cost of approximately USD 9150 per cancer-related DALY and an indirect cost of approximately USD 3890 per DALY [12]. The estimated total cost per cancer-related DALY corresponded, on average, to approximately 32% of the respective national gross domestic product per capita [12]. Collectively, the substantial epidemiological and socioeconomic burden of cancer underscores the need for therapeutic strategies that achieve durable tumor control while minimizing treatment-related morbidity.

The pivotal role of VEGF-mediated angiogenesis in tumor progression provided a strong rationale for the development of antiangiogenic therapies. As a result, VEGF-targeted agents have become important components of treatment strategies for a variety of malignancies, including colorectal cancer, renal cell carcinoma, hepatocellular carcinoma, non-small-cell lung cancer, thyroid cancer, and glioblastoma [9,10,11]. Therapeutic approaches include monoclonal antibodies, ligand traps, and small-molecule VEGF receptor tyrosine kinase inhibitors (VEGFR-TKIs), such as bevacizumab, sunitinib, sorafenib, pazopanib, axitinib, and lenvatinib. These agents suppress tumor angiogenesis, delay disease progression, and, in some settings, improve overall survival [9,10,11]. Furthermore, emerging evidence suggests that antiangiogenic therapies may enhance the effectiveness of immunotherapy and other targeted treatments through vascular normalization and modulation of the tumor microenvironment [10,11,12].

Despite their clinical success, VEGF-targeted therapies face significant challenges that limit their long-term efficacy. Resistance frequently develops through activation of alternative proangiogenic pathways and adaptive changes within the tumor microenvironment [11,12,13]. In addition, because VEGF signaling is essential for vascular and renal homeostasis, therapeutic inhibition is associated with cardiovascular and renal adverse effects, including hypertension, endothelial dysfunction, thromboembolic events, proteinuria, and kidney injury [12,13,14]. These toxicities may require dose modification or treatment discontinuation, ultimately affecting patient outcomes. Therefore, improving the therapeutic index of VEGF-targeted therapies remains a major clinical objective.

The complexity of VEGF biology reflects the coordinated actions of multiple VEGF ligands and receptors, each exhibiting distinct receptor-binding affinities, tissue distributions, and biological functions. This diversity allows precise regulation of angiogenesis, lymphangiogenesis, vascular permeability, immune-cell trafficking, and tissue remodeling under both physiological and pathological conditions. Moreover, individual VEGF family members contribute differentially to tumor vascularization, inflammation, metastasis, and therapeutic resistance, highlighting the importance of understanding ligand-specific signaling mechanisms.

Given the multifaceted roles of VEGF signaling in vascular biology, cancer progression, therapeutic response, and treatment-associated toxicity, a comprehensive understanding of this pathway remains essential. In this review, we summarize the biological functions of VEGF ligands, VEGF receptors, and their downstream signaling networks. We discuss the role of VEGF signaling in physiological angiogenesis and tumor progression, examine the clinical applications and limitations of current VEGF-targeted therapies, and highlight strategies aimed at improving therapeutic efficacy while minimizing cardiovascular and renal toxicity. Emphasis is placed on clinically established VEGFR-targeted approaches, representative quantitative evidence for VEGFR-TKIs, mechanisms of resistance, and current toxicity management. Emerging interventions, including EET-based therapies, soluble epoxide hydrolase inhibition, endothelin-directed strategies, and natural compounds, are discussed separately as investigational approaches whose clinical value requires further validation.

### Literature Search Strategy

A structured literature search was conducted to identify relevant publications addressing VEGF/VEGFR signaling, tumor angiogenesis and lymphangiogenesis, VEGF-targeted cancer therapies, therapeutic resistance, and cardiovascular and renal toxicities associated with VEGF pathway inhibition. Relevant peer-reviewed literature was identified using PubMed, Scopus, and Web of Science, supplemented by screening the reference lists of pertinent original studies and review articles. Search terms included combinations of “vascular endothelial growth factor,” “VEGF,” “VEGFR,” “VEGFR1,” “VEGFR2,” “VEGFR3,” “angiogenesis,” “lymphangiogenesis,” “cancer,” “VEGF-targeted therapy,” “VEGFR tyrosine kinase inhibitor,” “anti-VEGF therapy,” “therapeutic resistance,” “cardiovascular toxicity,” “hypertension,” “renal toxicity,” “proteinuria,” “nephrotoxicity,” “endothelin-1,” “epoxyeicosatrienoic acids,” “EET analogs,” and “soluble epoxide hydrolase.” The literature search included publications available through 30 September 2026. Studies were selected based on their relevance to the predefined scope of the review, including mechanistic investigations of VEGF/VEGFR signaling; preclinical and clinical studies of VEGF-targeted therapies; studies addressing therapeutic resistance or treatment-associated cardiovascular and renal toxicities; and reports evaluating established or emerging toxicity-mitigation strategies. Priority was given to peer-reviewed original research articles, clinical trials, systematic reviews, meta-analyses, and authoritative reviews that directly informed the molecular, therapeutic, or clinical aspects of the review. Seminal studies were retained where necessary to provide historical or mechanistic context, while recent publications were prioritized to reflect current advances in the field. Publications lacking direct relevance to the predefined scope of the review were excluded.

## 2. The Ligands of VEGF

**Figure 1:**
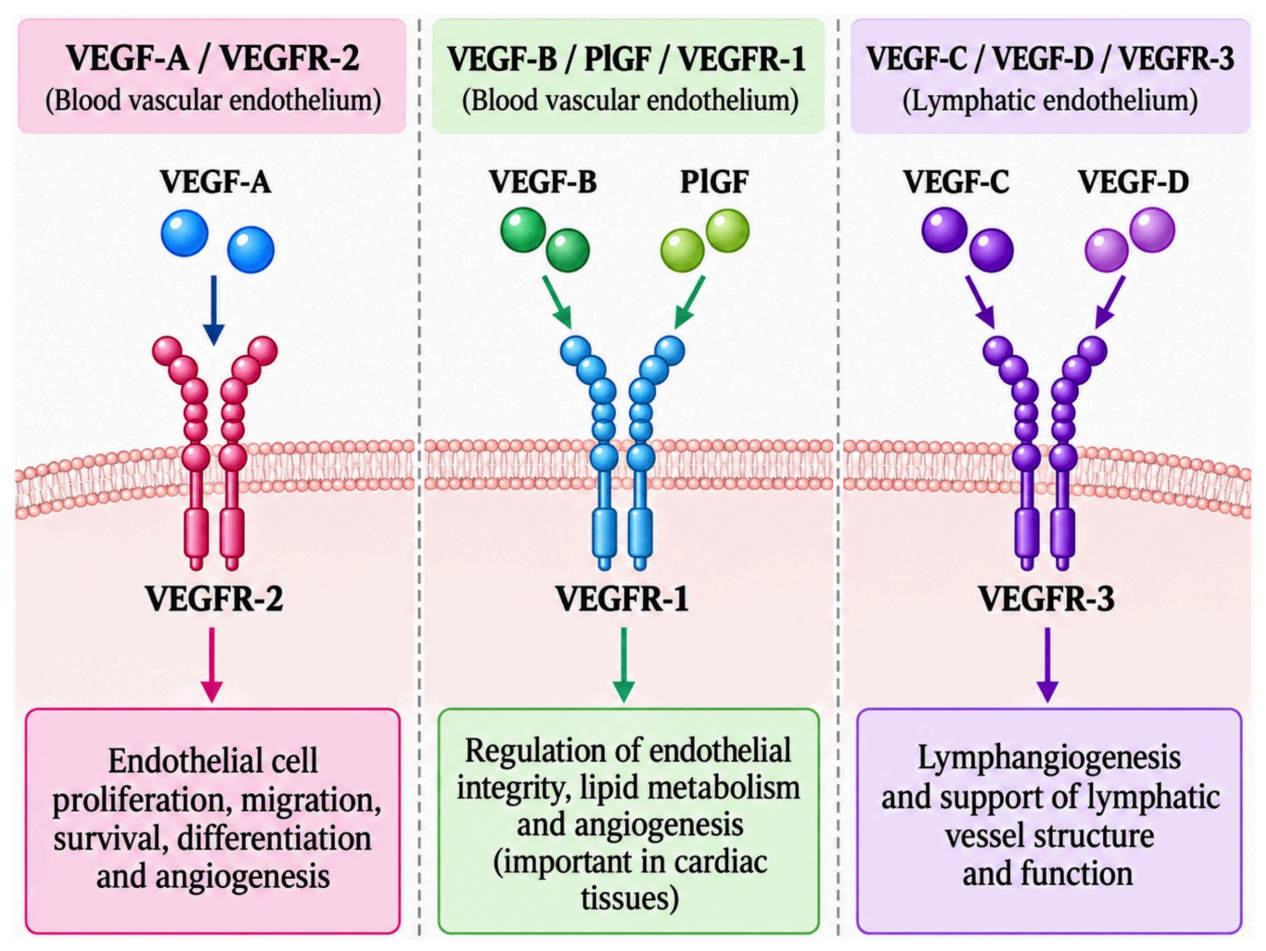
Receptor-specific functions of the VEGF family in vascular and lymphatic endothelial biology. Graphical elements used in the figure were created using BioRender (BioRender, Toronto, ON, Canada; BioRender; accessed [June 2026]; RRID: SCR_018361). Schematic overview of vascular endothelial growth factor (VEGF) family ligands and their principal receptor interactions. VEGF-A binds VEGFR-1 and VEGFR-2, with VEGFR-2 serving as the primary mediator of endothelial cell proliferation, migration, survival, vascular permeability, and angiogenesis. VEGF-B and placental growth factor (PlGF) preferentially bind VEGFR-1 and contribute to endothelial regulation and angiogenic signaling, whereas VEGF-B also supports metabolic and trophic functions in highly active tissues. VEGF-C and VEGF-D predominantly activate VEGFR-3, promoting lymphatic endothelial cell proliferation, migration, survival, and lymphangiogenesis. Mature VEGF-C and VEGF-D may additionally activate VEGFR-2.

The vascular endothelial growth factor (VEGF) family consists of five structurally related ligands: VEGF-A, VEGF-B, VEGF-C, VEGF-D, and placental growth factor (PlGF). These ligands regulate angiogenesis, lymphangiogenesis, vascular permeability, endothelial survival, and immune-cell recruitment through activation of VEGFR-1, VEGFR-2, and VEGFR-3. Although they share considerable structural homology, VEGF family members exhibit distinct receptor-binding preferences and biological functions that collectively regulate vascular and lymphatic homeostasis under physiological and pathological conditions, including cancer, chronic inflammation, and vascular disease [3,13]. In Figure 1, we schematically depict VEGF ligand–receptor interactions in vascular and lymphatic signaling.

VEGF-family ligands have also been characterized structurally by X-ray crystallography and related structural approaches, providing a molecular framework for understanding ligand dimerization and receptor recognition. Representative Protein Data Bank (PDB) structures are available for members of the VEGF family and for ligand–receptor complexes, including VEGF-A-containing structures (for example, PDB 1VPF), PlGF (PDB 1FZV), VEGF-B (PDB 2C7W), and processed VEGF-C in receptor-binding complexes (for example, PDB 4BSK). These structural resources complement biochemical studies by defining the conserved cystine-knot architecture and receptor-binding surfaces of VEGF ligands [10]. Because structural coverage differs among ligands, isoforms, maturation states, and complex compositions, individual PDB records should be interpreted in the context of the specific construct and experimental system used.

### 2.1. VEGF-A

VEGF-A is the principal regulator of angiogenesis and the best-characterized member of the VEGF family. Acting primarily through VEGFR-2 and, to a lesser extent, VEGFR-1, VEGF-A stimulates endothelial proliferation, migration, survival, vascular permeability, and neovascularization [1,14,15]. Activation of VEGFR-2 triggers the PI3K-AKT and PLCγ-PKC-MAPK pathways, which coordinate angiogenic growth and vascular remodeling [14,15]. Although VEGFR-1 binds VEGF-A with high affinity, its weaker kinase activity allows it to function largely as a regulatory receptor controlling VEGF availability while also contributing to inflammatory-cell recruitment and pathological angiogenesis [1,15,16,17]. In cancer, VEGF-A is frequently overexpressed in response to hypoxia, oncogenic signaling, and inflammatory stimuli, resulting in abnormal vascular growth that supports tumor survival, invasion, and metastasis [1,14]. Consequently, VEGF-A remains the primary target of most currently approved antiangiogenic therapies.

### 2.2. VEGF-B and PlGF

Unlike VEGF-A, VEGF-B and PlGF bind selectively to VEGFR-1 and perform more specialized regulatory functions. VEGF-B is expressed predominantly in metabolically active tissues, including the heart, skeletal muscle, and nervous system, where it supports endothelial survival, vascular maintenance, and metabolic regulation. Although its direct angiogenic activity is limited, VEGF-B may contribute to stabilization of existing vasculature and regulation of lipid metabolism [1,3,14,16]. PlGF plays a more prominent role in pathological angiogenesis and inflammation. Beyond its physiological functions in development, placentation, wound repair, and vascular remodeling, PlGF promotes communication among tumor, endothelial, and immune cells within the tumor microenvironment [3,13,16,17,18]. Through recruitment of monocytes and macrophages, PlGF enhances inflammatory signaling, angiogenesis, and metastatic progression [6,19]. Because PlGF expression is generally low under normal conditions, selective inhibition of PlGF has attracted interest as a strategy to suppress pathological angiogenesis while minimizing disruption of physiological vascular function [3,18,20].

### 2.3. VEGF-C and VEGF-D

VEGF-C and VEGF-D are the principal mediators of lymphangiogenesis and signal predominantly through VEGFR-3, although both can activate VEGFR-2 under specific conditions [3,20]. Through stimulation of VEGFR-3, these ligands promote lymphatic endothelial proliferation, migration, survival, and vessel remodeling, thereby maintaining lymphatic fluid balance, immune surveillance, and lipid transport [3,20]. In cancer, increased VEGF-C and VEGF-D expression promotes tumor-associated lymphangiogenesis and facilitates metastatic dissemination via the lymphatic system [6,20,21]. Elevated VEGF-C levels are strongly associated with lymphatic metastasis in several malignancies, including breast and prostate cancers [3,7,18]. Experimental inhibition of the VEGF-C/VEGFR-3 pathway suppresses lymphatic vessel growth and reduces metastatic spread, establishing this signaling axis as an important therapeutic target for preventing lymphatic dissemination [3,7,18,20].

Collectively, VEGF ligands exert distinct but complementary effects through selective VEGFR engagement: VEGF-A predominantly drives angiogenesis through VEGFR2, VEGF-B and PlGF modulate VEGFR1-dependent vascular and inflammatory responses, and VEGF-C/VEGF-D primarily regulate lymphangiogenesis through VEGFR3 [3,13,20]. These ligand–receptor preferences provide the biological framework for the receptor activation and signaling mechanisms discussed below.

## 3. Mechanisms of VEGF Signaling and VEGFR-Mediated Signal Transduction

Vascular endothelial growth factor (VEGF) regulates angiogenesis, lymphangiogenesis, vascular permeability, endothelial survival, and tissue adaptation through activation of vascular endothelial growth factor receptors (VEGFRs), a family of receptor tyrosine kinases comprising VEGFR1, VEGFR2, and VEGFR3 [3,6,14,21]. These receptors function as molecular signaling hubs that convert extracellular VEGF stimulation into coordinated intracellular responses governing vascular development, remodeling, and homeostasis. The major VEGF ligands, their corresponding receptors, and associated biological functions are summarized in Table 1.

### 3.1. Structure and Activation of VEGFRs

VEGFRs share a conserved structural organization consisting of an extracellular ligand-binding region containing seven immunoglobulin-like domains, a single transmembrane segment, and an intracellular tyrosine kinase domain [1,14,16,21]. Binding of VEGF ligands promotes receptor dimerization and conformational changes that bring intracellular kinase domains into proximity, resulting in receptor activation and transphosphorylation of specific tyrosine residues [14,23,24].

Ligand specificity determines receptor activation and downstream biological responses. VEGF-A primarily activates VEGFR2, whereas VEGF-C and VEGF-D preferentially activate VEGFR3 [23,25,27]. Signaling activity is further modulated by co-receptors and accessory molecules, including neuropilin-1 (NRP1), neuropilin-2 (NRP2), and heparan sulfate proteoglycans, which stabilize ligand–receptor interactions and promote receptor clustering [1,14,16,23].

Upon activation, phosphorylated tyrosine residues serve as docking platforms for adaptor proteins and signaling intermediates that initiate downstream transduction pathways. The pattern and extent of receptor phosphorylation determine the magnitude and specificity of endothelial responses, including proliferation, migration, survival, permeability, and vascular remodeling [23,28,29]. Tight regulation of these phosphorylation events is essential because excessive or prolonged receptor activation can disrupt vascular homeostasis and contribute to pathological vascular remodeling [16,24,28,32,39,40].

### 3.2. Major Downstream Signaling Pathways

Activated VEGFRs stimulate several interconnected intracellular signaling pathways that collectively regulate endothelial function and vascular adaptation. Among these, the MAPK-ERK, PI3K-AKT, and PLCγ pathways represent the principal downstream effectors of VEGF signaling [3,30,32,40].

#### 3.2.1. MAPK-ERK Signaling

The MAPK-ERK pathway is a major regulator of endothelial proliferation and angiogenic growth. Following VEGFR activation, recruitment of adaptor proteins initiates the Ras-Raf-MEK-ERK signaling cascade, culminating in activation of transcriptional programs that promote cell-cycle progression, endothelial expansion, migration, and angiogenic sprouting [29,32]. Beyond its proliferative function, ERK signaling contributes to cytoskeletal remodeling and angiogenic gene expression required for vascular development and tissue repair. While transient pathway activation supports physiological angiogenesis, sustained ERK signaling contributes to pathological angiogenesis, tumor vascularization, and chronic inflammatory vascular remodeling [30,32,39,41].

#### 3.2.2. PI3K-AKT Signaling

The PI3K-AKT pathway serves as a central regulator of endothelial survival, vascular stability, and adaptive stress responses [41,42,43,44]. Activation of PI3K generates phosphatidylinositol-3,4,5-trisphosphate, leading to recruitment and activation of AKT. Downstream signaling suppresses apoptosis and enhances endothelial resistance to oxidative stress, inflammation, and hypoxia. One of the most important consequences of AKT activation is phosphorylation of endothelial nitric oxide synthase (eNOS), resulting in increased nitric oxide production that supports vasodilation, endothelial migration, tissue perfusion, and vascular integrity [23,43,44,45]. In addition, PI3K-AKT signaling contributes to maintenance of endothelial barrier function and microvascular homeostasis. Dysregulation of this pathway has been implicated in endothelial dysfunction, pathological vascular leakage, diabetic retinopathy, and other vascular disorders [45,46].

#### 3.2.3. PLCγ Signaling

PLCγ signaling regulates endothelial migration, cytoskeletal dynamics, vascular permeability, and angiogenic sprouting [30,44]. Recruitment of PLCγ to activated VEGFRs results in hydrolysis of phosphatidylinositol-4,5-bisphosphate, generating inositol trisphosphate (IP3) and diacylglycerol (DAG) [23,28,29,30]. These second messengers stimulate intracellular calcium mobilization and activate protein kinase C, thereby coordinating endothelial motility, tip-cell behavior, and vascular remodeling. PLCγ signaling also regulates endothelial junctional dynamics and transient increases in vascular permeability required for tissue repair and adaptation. However, excessive pathway activation can promote pathological vascular leakage and abnormal angiogenesis in inflammatory diseases, retinal disorders, and cancer [3,23,44].

### 3.3. Integration of VEGFR Signaling Networks

Although MAPK-ERK, PI3K-AKT, and PLCγ pathways regulate distinct cellular processes, they function as components of a highly integrated signaling network that collectively governs endothelial proliferation, migration, survival, permeability, nitric oxide production, and vascular remodeling [42,43,44,45,46]. Extensive crosstalk occurs through shared adaptor proteins, co-receptor interactions, receptor trafficking pathways, and intracellular feedback mechanisms [18,44]. For example, PLCγ-mediated calcium signaling influences AKT-dependent eNOS activation, whereas protein kinase C signaling enhances ERK-mediated transcriptional responses, linking migratory, survival, and proliferative programs into a coordinated angiogenic response [42,43,44].

This signaling integration allows VEGFRs to generate context-dependent responses during embryonic development, tissue repair, lymphangiogenesis, and adaptation to hypoxic stress [3,18,23,44]. Under physiological conditions, balanced pathway activation promotes vascular homeostasis and adaptive remodeling. Conversely, persistent or dysregulated signaling contributes to pathological angiogenesis, lymphangiogenesis, vascular leakage, therapeutic resistance, chronic inflammation, and tumor progression [3,18,23,44,45,46].

Collectively, VEGFR signaling functions as a dynamic regulatory network that translates extracellular VEGF stimulation into coordinated vascular responses under both physiological and pathological conditions. Although VEGFR family members share common downstream signaling pathways, they differ substantially in ligand specificity, receptor expression patterns, signaling potency, intracellular trafficking, and biological function. Among these receptors, VEGFR2 serves as the principal mediator of VEGF-driven angiogenic signaling and therefore warrants individual consideration. The integrated relationships among receptor activation, MAPK-ERK, PI3K-AKT-eNOS, and PLCγ-PKC signaling and their principal endothelial outcomes are summarized schematically in Figure 1 and in the pathway-focused illustrations used throughout this review.

## 4. VEGFR2: The Central Mediator of Angiogenic Signaling

VEGFR2 (KDR in humans; Flk1 in mice) is the principal receptor responsible for VEGF-driven angiogenesis and the dominant signaling mediator within the VEGF receptor family [3,16]. Activated primarily by VEGF-A, particularly VEGF-A165, VEGFR2 regulates endothelial proliferation, migration, survival, vascular permeability, and neovascularization during development, tissue repair, and pathological angiogenesis [24,29,31,33,39,40]. Compared with VEGFR1, which frequently functions as a regulatory or decoy receptor, and VEGFR3, which primarily regulates lymphatic vascular biology, VEGFR2 serves as the primary signaling receptor controlling blood vascular growth and remodeling [16,31,38,47].

Structurally, VEGFR2 contains seven extracellular immunoglobulin-like domains, a single transmembrane region, and an intracellular split tyrosine kinase domain. Ligand-induced dimerization and phosphorylation initiate the signaling networks summarized in Section 3.2 [29,31]. In this receptor-specific context, VEGFR2 is distinguished by its dominant role in blood-vessel growth, endothelial nitric oxide production, permeability, and adaptive angiogenesis [29,30,32,35,37,44,45,48,49]. Its activity is further shaped by coreceptors and receptor trafficking, which regulate the magnitude, location, and duration of signaling.

### 4.1. Coreceptor Regulation of VEGFR2 Signaling

VEGFR2 activity is strongly influenced by coreceptors and accessory molecules that regulate ligand binding, receptor activation, trafficking, and signal amplification [3,31]. Among these, neuropilin-1 (NRP1) is the most extensively characterized. NRP1 binds VEGF-A165 and promotes formation of VEGF-A165-NRP1-VEGFR2 complexes, thereby enhancing receptor activation and downstream angiogenic signaling [5,37]. Beyond signal amplification, NRP1 regulates receptor internalization and endosomal signaling, providing spatial and temporal control of endothelial responses required for vessel branching, migration, and angiogenic sprouting [3,18,49].

Heparan sulfate proteoglycans (HSPGs) further modulate VEGFR2 signaling by concentrating VEGF ligands within the extracellular matrix and facilitating efficient ligand–receptor interactions [15]. Through regulation of VEGF bioavailability and gradient formation, HSPGs contribute to endothelial guidance and vascular patterning [3,32]. Additional interactions with integrins, cadherins, and other membrane-associated proteins integrate VEGFR2 signaling with extracellular matrix cues and cell–cell adhesion pathways. These interactions enhance endothelial responsiveness to VEGF and coordinate migration, survival, vascular remodeling, and tissue-specific angiogenic responses [29,31,44]. Collectively, these accessory molecules fine-tune VEGFR2 signaling to meet developmental, physiological, and pathological requirements.

### 4.2. Receptor Trafficking and Sustained VEGFR2 Signaling

Receptor trafficking is a major determinant of VEGFR2 signaling strength, duration, and biological specificity [29,50,51]. Following VEGF-A binding, VEGFR2 undergoes clathrin-mediated endocytosis and continues signaling from intracellular compartments known as signaling endosomes rather than being immediately degraded [15,23,50,51]. Sustained signaling from these compartments supports prolonged ERK- and AKT-dependent responses required for endothelial survival, angiogenic gene expression, vascular sprouting, and vessel maturation [23,50,51].

The fate of internalized VEGFR2 is controlled through tightly regulated trafficking pathways. Receptors may be recycled to the plasma membrane, restoring endothelial responsiveness to VEGF stimulation, or directed toward lysosomal degradation, thereby terminating signaling [26,29,52]. This balance plays a critical role in determining the magnitude and duration of angiogenic responses.

Multiple trafficking regulators influence VEGFR2 availability and signaling competence. Rab GTPases, RABEP2, S100A6, and ubiquitination pathways regulate receptor transport, recycling, membrane delivery, and degradation, thereby maintaining appropriate receptor levels and preserving endothelial responsiveness to VEGF [26,29,44,53,54]. Coreceptors such as NRP1 further influence receptor internalization and endosomal sorting, promoting sustained angiogenic signaling and endothelial migration [26,44].

Alterations in VEGFR2 trafficking have important pathological consequences. Excessive receptor recycling may contribute to persistent angiogenesis, tumor progression, and vascular leakage, whereas enhanced degradation may impair adaptive angiogenesis and tissue repair after ischemic injury [26,29,55]. Consequently, proteins regulating VEGFR2 trafficking have emerged as promising therapeutic targets capable of modulating angiogenesis without directly inhibiting receptor kinase activity [26,32,40].

Collectively, VEGFR2 integrates extracellular VEGF stimulation with intracellular signaling pathways, coreceptor networks, and trafficking mechanisms that govern endothelial growth, migration, survival, permeability, and vascular remodeling [3,15,23,29,31]. These regulatory processes are essential for vascular homeostasis, whereas their dysregulation contributes to tumor angiogenesis, retinal disease, vascular leakage, and cardiovascular pathology [26,29,32,55]. Despite the central role of VEGFR2 in blood vessel biology, maintenance of tissue homeostasis also depends on the lymphatic vasculature. Consequently, understanding VEGFR signaling requires consideration of VEGFR3, the principal regulator of lymphangiogenesis and lymphatic vascular remodeling.

## 5. VEGFR3: The Central Regulator of Lymphangiogenic Signaling

VEGFR3 (FLT4) is the principal receptor governing lymphangiogenesis and the dominant signaling mediator within the lymphatic vascular system [26,27,56,57,58]. Activated primarily by VEGF-C and VEGF-D, VEGFR3 regulates lymphatic endothelial cell proliferation, migration, survival, differentiation, and vessel maturation, thereby controlling the formation and maintenance of functional lymphatic networks [26,27,56]. While VEGFR2 predominantly coordinates blood vascular angiogenesis, VEGFR3 serves as the primary signaling receptor responsible for lymphatic vessel development, remodeling, and homeostasis [26,27,57].

Structurally, VEGFR3 shares the conserved organization characteristic of the VEGFR family, comprising seven extracellular immunoglobulin-like domains, a single transmembrane region, and an intracellular tyrosine kinase domain [21,26]. Ligand binding induces receptor dimerization and autophosphorylation of intracellular tyrosine residues, initiating signaling pathways that regulate lymphatic endothelial growth and function [26,57,58]. Genetic studies have demonstrated that VEGFR3 is indispensable for embryonic lymphatic vascular development, as disruption of VEGFR3 signaling results in severe lymphatic defects and embryonic lethality [26,57].

Through activation of downstream signaling pathways including PI3K-AKT, MAPK-ERK, SRC family kinases, and PLCγ-dependent mechanisms, VEGFR3 promotes lymphatic endothelial survival, migration, sprouting, and vessel maturation [26,57,58,59]. Beyond its established role in lymphangiogenesis, VEGFR3 contributes to regulation of immune-cell trafficking, interstitial fluid homeostasis, lipid absorption, inflammatory resolution, and tissue regeneration [26,27,56,57]. Increasing evidence also demonstrates that VEGFR3 participates in pathological processes including tumor-associated lymphangiogenesis, lymph node metastasis, chronic inflammation, and fibrotic remodeling [26,27,56,57,58]. Consequently, VEGFR3 has emerged as an important therapeutic target in oncology, inflammatory disease, and disorders of lymphatic vascular function.

### 5.1. Regulation of VEGFR3 Activation and Lymphatic Signaling

VEGFR3 signaling is tightly regulated by ligand availability, receptor processing, co-receptor interactions, and extracellular matrix components that collectively determine signaling magnitude and biological outcomes [26,56,57,58]. VEGF-C represents the dominant physiological activator of VEGFR3 and requires proteolytic processing to achieve maximal receptor-binding affinity and signaling potency [26,56]. VEGF-D undergoes similar maturation-dependent activation and contributes to lymphatic remodeling in both physiological and pathological settings [26,27].

Several accessory molecules further modulate VEGFR3 activity. Neuropilin-2 (NRP2) functions as a major co-receptor that enhances VEGF-C-mediated VEGFR3 signaling, promoting lymphatic endothelial migration, sprouting, and vessel branching [57,59]. Heparan sulfate proteoglycans and extracellular matrix components also influence ligand distribution and receptor accessibility, thereby regulating lymphatic vessel patterning and guidance [26,56].

In addition to homodimer formation, VEGFR3 can interact with VEGFR2, generating receptor heterodimers that integrate lymphangiogenic and angiogenic signaling pathways [58,59,60,61,62]. This interaction has emerged as an important mechanism coordinating blood vascular and lymphatic vascular remodeling during development, tissue repair, inflammation, and tumor progression. Through these diverse regulatory mechanisms, VEGFR3 signaling is precisely tuned to match physiological demands while preventing excessive lymphatic growth and dysfunction.

### 5.2. Receptor Trafficking and Sustained VEGFR3 Signaling

As observed with VEGFR2, receptor trafficking is increasingly recognized as an important regulator of VEGFR3 signaling duration, intensity, and biological specificity [26,58,60]. Following ligand binding, VEGFR3 undergoes endocytosis and continues signaling from intracellular compartments, enabling prolonged activation of signaling pathways involved in lymphatic endothelial survival, migration, and vessel maturation [58,60].

The balance between receptor recycling and degradation contributes significantly to lymphatic responsiveness. Recycling pathways restore receptor availability at the cell surface, whereas lysosomal degradation limits signaling duration and prevents excessive lymphangiogenic activity [26,58]. These trafficking processes provide a mechanism through which lymphatic endothelial cells dynamically adjust their sensitivity to VEGF-C and VEGF-D stimulation.

Emerging evidence suggests that dysregulation of VEGFR3 trafficking may contribute to pathological lymphangiogenesis and disease progression. Enhanced receptor recycling can promote sustained lymphatic expansion and metastatic dissemination, whereas impaired signaling may compromise lymphatic repair and fluid drainage [26,56,58,60]. Consequently, proteins regulating VEGFR3 trafficking have attracted increasing attention as potential therapeutic targets capable of modulating lymphangiogenesis without directly inhibiting receptor kinase activity.

### 5.3. VEGFR2-VEGFR3 Crosstalk and Coordinated Vascular Remodeling

Although VEGFR2 and VEGFR3 were historically viewed as separate regulators of blood and lymphatic vessels, respectively, they can form heterodimeric receptor complexes that integrate angiogenic and lymphangiogenic signaling [58,59,60,61,62]. VEGF-C and VEGF-D can engage both receptors under specific conditions, enabling coordinated activation of PI3K-AKT, MAPK-ERK, and PLCγ-PKC pathways and influencing endothelial migration, survival, permeability, receptor trafficking, and adaptive vascular remodeling [23,34,36,58,59,60,61,62].

This crosstalk is relevant during development, tissue repair, inflammation, and cancer, where blood-vessel and lymphatic remodeling often occur simultaneously [58,59,60,61,62]. In tumors, coordinated VEGFR2-VEGFR3 signaling can support vascularization, lymphangiogenesis, local invasion, and metastatic dissemination, while VEGF-C/VEGFR3 activity may also provide an alternative remodeling pathway during VEGF-A/VEGFR2 inhibition [26,27,56,57,58,59,60,61,62]. These observations support considering VEGFR2 and VEGFR3 as interconnected components of the VEGF network rather than fully independent signaling systems.

The biological importance of VEGFR2-VEGFR3 interactions is increasingly recognized in cancer, where coordinated angiogenic and lymphangiogenic remodeling facilitates tumor growth, local invasion, and metastatic spread. Similar mechanisms contribute to chronic inflammatory diseases, retinal vascular disorders, and tissue fibrosis, underscoring the broader significance of receptor crosstalk in vascular pathology [26,56,57,58,59,60,61,62].

Collectively, VEGFR3 integrates extracellular VEGF-C and VEGF-D stimulation with intracellular signaling networks, co-receptor interactions, and receptor trafficking mechanisms that regulate lymphatic endothelial growth, migration, survival, and vessel remodeling [26,27,56,57,58,59,60]. Furthermore, dynamic interactions between VEGFR3 and VEGFR2 coordinate blood vascular and lymphatic vascular responses across diverse physiological and pathological settings [58,59,60,61,62]. These regulatory processes are essential for maintaining fluid balance, immune surveillance, tissue repair, and vascular homeostasis, whereas their dysregulation contributes to lymphedema, chronic inflammation, tumor-associated lymphangiogenesis, and metastatic disease [26,27,56,57,58,59,60,61,62].

## 6. VEGF Inhibition: Therapeutic Applications, Resistance, and Future Directions

VEGF signaling is a central regulator of angiogenesis, vascular remodeling, and tumor progression, making it one of the most extensively targeted pathways in modern oncology [3,6,18]. VEGF overexpression promotes endothelial proliferation, migration, vascular permeability, and formation of abnormal tumor vasculature that supports tumor growth, metastasis, and immune evasion [3,6,18]. Consequently, multiple therapeutic strategies have been developed to inhibit VEGF signaling, including receptor tyrosine kinase inhibitors (TKIs), monoclonal antibodies, and VEGF ligand traps [3,4,24,61,62]. Collectively, these agents have transformed the treatment of numerous angiogenesis-dependent malignancies and established angiogenesis as a clinically actionable hallmark of cancer [24,61,62,63]. Despite substantial clinical success, the long-term efficacy of VEGF-targeted therapies is frequently limited by intrinsic and acquired resistance, activation of compensatory angiogenic pathways, tumor microenvironment adaptation, and treatment-related toxicities [24,61,62]. Because VEGF signaling interacts extensively with HIF, FGF, PDGF, HGF, angiopoietin, PI3K/AKT, MAPK/ERK, mTOR, and immune-regulatory pathways, inhibition of VEGF alone often triggers adaptive mechanisms that restore vascular growth and support tumor survival [57,64,65,66,67,68,69,70].

### 6.1. VEGFR Tyrosine Kinase Inhibitors

VEGFR-TKIs are small-molecule inhibitors that block the intracellular kinase activity of VEGF receptors, particularly VEGFR2, thereby inhibiting pathways required for endothelial proliferation, angiogenesis, and tumor vascular maintenance [3,4,24,48,60]. Agents including sunitinib, sorafenib, pazopanib, axitinib, lenvatinib, regorafenib, and cabozantinib are widely used in renal cell carcinoma, hepatocellular carcinoma, thyroid cancer, gastrointestinal stromal tumors, and neuroendocrine tumors [48,60,71,72,73]. Unlike ligand-targeted therapies, most VEGFR-TKIs inhibit multiple receptor tyrosine kinases, including PDGFR, FGFR, KIT, RET, MET, and AXL, thereby targeting several angiogenic and survival pathways simultaneously [63,74]. This broader activity contributes to their antitumor efficacy and may promote transient vascular normalization that enhances delivery of chemotherapy and immunotherapy [64,75].

### 6.2. Monoclonal Antibodies and VEGF Ligand Traps

Monoclonal antibodies and VEGF ligand traps inhibit VEGF signaling extracellularly by preventing ligand–receptor interactions [64,67,76]. Bevacizumab, a humanized antibody targeting VEGF-A, has demonstrated clinical benefit in several solid malignancies [76,77,78,79,80,81]. Aflibercept (ziv-aflibercept in oncology) is a systemic VEGF ligand trap composed of VEGFR1 and VEGFR2 extracellular binding domains fused to human IgG Fc; it sequesters VEGF-A and PlGF before receptor activation [82,83,84,85]. Its oncological use should be distinguished from intravitreal aflibercept formulations used for retinal disease; ophthalmological efficacy data are not evidence of anticancer efficacy. VEGF inhibition may also reduce immunosuppressive features of the tumor microenvironment, providing a rationale for combinations with immune checkpoint inhibitors [80,81,86,87,88,89].

### 6.3. VEGF-Targeted Therapy Combined with Immune Checkpoint Inhibition

The biological interaction between angiogenesis and immune suppression provides a rationale for combining VEGF/VEGFR-directed therapy with immune checkpoint inhibition. VEGF blockade may reduce immunosuppressive features of the tumor microenvironment and improve immune-cell access, while checkpoint inhibition restores antitumor T-cell activity [80,81,86,87,88,89]. Clinical studies have established VEGF-targeted/immunotherapy combinations in several cancer settings, although efficacy and toxicity are regimen- and disease-specific. These combinations therefore require attention to patient selection and overlapping vascular, renal, and immune-mediated adverse effects.

### 6.4. Resistance to VEGF-Targeted Therapy

Resistance remains the principal barrier to durable VEGF-targeted therapy [57,65,66,67,86,87,90]. VEGF inhibition frequently increases intratumoral hypoxia, resulting in HIF-mediated upregulation of alternative proangiogenic pathways, including FGF, PDGF, HGF, angiopoietins, inflammatory cytokines, and other survival signals [64,65,66,75,90]. Recruitment of tumor-associated macrophages, mast cells, and myeloid-derived suppressor cells further contributes to restoration of tumor vascularization and therapeutic escape [66,67]. Additional resistance mechanisms include activation of PI3K/AKT and MAPK/ERK signaling, metabolic reprogramming, changes in drug transport systems, and adaptation of the tumor microenvironment [65,66,67,68,69,70]. Emerging evidence also suggests that VEGF-C/VEGFR3 signaling may provide an alternative vascular remodeling pathway that contributes to resistance against VEGF-A- and VEGFR2-directed therapies [26,27,29].

### 6.5. Adverse Effects of VEGF Inhibition

Because VEGF signaling is essential for physiological vascular homeostasis, systemic inhibition frequently produces dose-limiting toxicities [65,66,88,89,91,92]. Common adverse effects include hypertension, proteinuria, impaired wound healing, hemorrhage, thromboembolic events, cardiovascular dysfunction, and renal injury [64,65,66,67,85,87,88,89]. Although the toxicity profiles of TKIs, monoclonal antibodies, and ligand traps differ somewhat, they share a common biological foundation arising from suppression of VEGF-dependent endothelial survival, nitric oxide production, microvascular maintenance, and vascular repair mechanisms [24,34,62]. These toxicities often require dose adjustment, temporary treatment interruption, or multidisciplinary clinical management.

### 6.6. Future Directions and Clinical Implications

The clinical success of VEGF-targeted therapies marks a major advance in translating vascular biology into effective anticancer treatment. VEGFR tyrosine kinase inhibitors, monoclonal antibodies, and VEGF ligand traps have improved therapeutic outcomes across multiple malignancies by suppressing tumor angiogenesis and disrupting the vascular support required for tumor growth and progression [24,62,63,64,65,66,67,68,69,70,76,77,78,79,80,81,82,83,84,85,86,87,88,89,91]. However, the durability of these responses is often limited by the complexity, redundancy, and adaptability of angiogenic signaling. Tumors can circumvent VEGF pathway inhibition by activating alternative proangiogenic pathways, engaging compensatory signaling networks, remodeling the microenvironment, and exploiting intratumoral heterogeneity [32,57,64,65,66,67,68]. Angiogenesis is also closely integrated with immune regulation, stromal interactions, metabolic adaptation, and tumor-cell survival, further contributing to therapeutic resistance. These limitations underscore the need to move beyond uniform pathway inhibition toward biomarker-informed and mechanistically guided treatment strategies.

A major priority is therefore the development of predictive and pharmacodynamic biomarkers capable of identifying patients most likely to benefit from VEGF/VEGFR-targeted therapy, monitoring treatment response, and detecting emerging resistance or toxicity [32,66,67,85]. Candidate biomarkers encompass circulating, tissue-based, molecular, and imaging-derived measures, including VEGF-family ligands, soluble VEGF receptors, vascular and inflammatory mediators, circulating microRNAs, transcriptomic signatures, tumor VEGF/VEGFR expression, hypoxia-related molecular profiles, and functional measures of tumor perfusion and vascularity [32,66,67,68,85]. Integration of these complementary biomarkers may ultimately provide a more informative assessment of angiogenic pathway activity than any single marker alone. Nevertheless, most remain investigational and require prospective validation within defined tumor types, therapeutic regimens, and clinical contexts before incorporation into routine patient stratification or treatment monitoring.

Emerging multi-omic and spatial technologies offer additional opportunities to resolve the biological heterogeneity underlying therapeutic response. Genomics, transcriptomics, proteomics, metabolomics, spatial transcriptomics, single-cell sequencing, and systems-level analyses may help define tumor-, vascular-, and patient-specific determinants of sensitivity and resistance. Their integration with functional imaging, circulating biomarkers, and clinical variables could support more precise selection and longitudinal monitoring of VEGF/VEGFR-targeted therapy. Such approaches may also guide rational combination strategies designed to overcome compensatory signaling while limiting unnecessary toxicity. These include biomarker-guided combinations of VEGF-targeted agents with immunotherapies and inhibitors of complementary signaling pathways, including PI3K/AKT/mTOR and MAPK/ERK [26,32,68,69,70].

Alongside these molecular approaches, emerging nanoscale and mechanobiological technologies may reveal dimensions of VEGF/VEGFR dysregulation that are not readily captured by conventional bulk assays. Atomic force microscopy (AFM), for example, combines nanoscale topographic imaging with force spectroscopy, molecular-recognition measurements, and nanoindentation, allowing structural and mechanical properties of biomolecules, membranes, cells, and tissues to be interrogated at high spatial resolution [69,70]. Advanced modalities further extend these capabilities: high-speed AFM can resolve dynamic biomolecular processes, whereas AFM-IR couples’ nanoscale morphological analysis with chemical characterization. Collectively, these technologies provide an emerging framework for linking molecular signaling to changes in membrane organization, receptor-associated interactions, cellular mechanics, adhesion, and other biophysical phenotypes relevant to cancer biology.

The potential relevance of this approach to VEGFR signaling is demonstrated by experimental studies of hypoxia-induced endothelial dysfunction. Prolonged hypoxia increased endothelial VEGFR-1 expression while inducing senescence-associated morphological and nanomechanical changes, including cellular flattening, altered membrane roughness, and progressively increased membrane stiffness measured by AFM [70]. VEGFR-1 knockdown attenuated these hypoxia-associated changes and substantially reduced the increase in membrane stiffness, directly connecting altered VEGFR-1 signaling with an endothelial nanomechanical phenotype. Mechanistically, hypoxia also reduced YAP-1 expression, whereas VEGFR-1 knockdown restored YAP-1 expression and delayed endothelial senescence, supporting functional crosstalk between VEGFR-1 signaling, cellular mechanics, and the mechanosensitive Hippo/YAP pathway [69,70]. These observations illustrate how nanoscale measurements can complement molecular and imaging approaches by providing biophysical readouts of dysregulated VEGFR signaling.

Nevertheless, the translational potential of nanoscale technologies should be interpreted cautiously. AFM-based approaches remain affected by challenges involving sample preparation and standardization, measurement reproducibility, analysis under physiologically relevant conditions, integration with complementary imaging platforms, and processing of complex multidimensional datasets. Accordingly, their most immediate value lies in mechanistic investigation and biomarker discovery rather than established clinical prognosis or routine therapeutic monitoring. Future integration with correlative imaging, automated analysis, chemometrics, machine learning, and other computational approaches may improve reproducibility and facilitate identification of nanoscale signatures associated with disease states or therapeutic responses.

Importantly, future strategies must also account for the dual biological role of VEGF signaling. VEGF is not solely a mediator of pathological tumor angiogenesis; it is also essential for physiological vascular and organ homeostasis. VEGF signaling supports endothelial survival, vascular integrity, nitric oxide production, microvascular maintenance, tissue perfusion, and adaptive responses to injury and hypoxia [6,24,34,36,62]. Thus, therapeutic VEGF inhibition creates an inherent biological trade-off: suppression of tumor vascularization may occur at the expense of normal endothelial and microvascular function.

Disruption of physiological VEGF signaling can impair endothelial function and nitric oxide bioavailability while promoting vasoconstriction, microvascular rarefaction, oxidative stress, inflammation, tissue hypoxia, mitochondrial dysfunction, and fibrotic remodeling [47,93,94,95,96,97,98,99,100,101,102,103,104,105]. These disturbances contribute to clinically important cardiovascular and renal toxicities, including hypertension, thrombotic events, cardiac dysfunction, proteinuria, glomerular injury, and chronic kidney impairment. Therefore, optimization of VEGF-targeted therapy should not be defined solely by the magnitude of angiogenic suppression. Rather, future therapeutic strategies should seek to achieve durable control of pathological angiogenesis while minimizing disruption of the physiological VEGF signaling required for vascular, cardiovascular, and renal homeostasis.

Ultimately, the next generation of VEGF/VEGFR-directed therapy will require integration of tumor biology, biomarker-guided patient selection, mechanisms of resistance, emerging multidimensional technologies, and treatment-related vascular toxicity. A more precise understanding of when, where, and in which cellular compartments VEGF/VEGFR signaling should be inhibited may enable development of more selective and durable therapeutic strategies. Equally important will be the identification of biomarkers that distinguish effective antitumor vascular inhibition from emerging injury to normal vasculature and target organs. Achieving this balance between sustained anticancer efficacy and preservation of cardiovascular and renal homeostasis represents a central challenge for future VEGF-directed therapy. The following section therefore examines the cardiovascular and renal consequences of VEGF pathway inhibition and the molecular mechanisms underlying these clinically important toxicities.

## 7. Consequences of VEGF Inhibition

Although VEGF-targeted therapies have significantly improved outcomes in several angiogenesis-dependent malignancies, VEGF signaling is essential not only for pathological angiogenesis but also for maintaining physiological vascular and renal homeostasis. In mature tissues, VEGF supports endothelial survival, vascular integrity, microvascular architecture, and tissue perfusion [6,24,34,36,62]. Consequently, systemic inhibition of VEGF signaling can disrupt these homeostatic mechanisms and produce clinically significant cardiovascular and renal adverse effects that may limit treatment tolerability and continuity [6,34,36,76].

Many of these toxicities arise from suppression of VEGFR2-mediated signaling pathways, particularly PI3K-AKT, MAPK-ERK, and PLCγ-PKC, which collectively regulate endothelial survival, nitric oxide (NO) production, vascular homeostasis, and adaptive microvascular remodeling [24,34,61]. Inhibition of these pathways promotes interconnected pathological responses, including endothelial dysfunction, reduced NO bioavailability, vasoconstriction, oxidative stress, microvascular rarefaction, tissue hypoxia, inflammation, and fibrotic remodeling. These disturbances provide a mechanistic basis for major clinical manifestations of VEGF pathway inhibition, including hypertension, cardiovascular dysfunction, proteinuria, glomerular injury, and progressive renal impairment.

**Figure 2:**
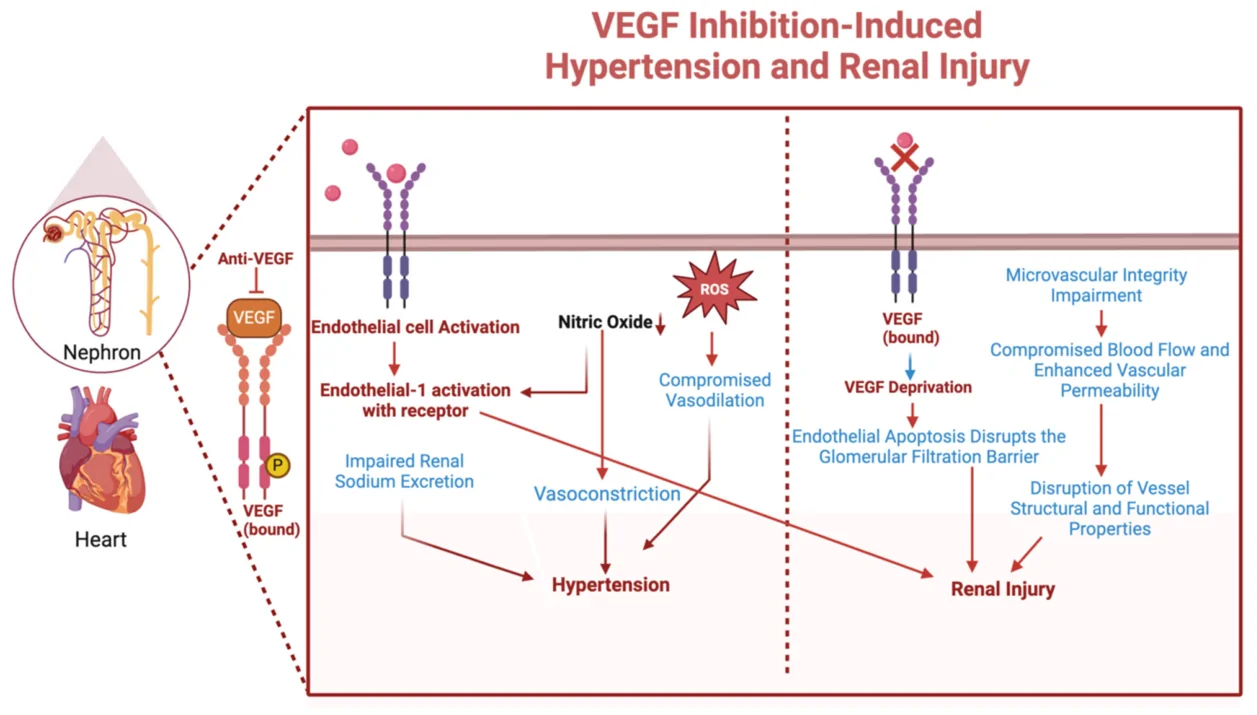
Molecular mechanisms of VEGF inhibitor-induced hypertension and renal injury. Graphical elements used in the figure were created using BioRender (BioRender, Toronto, ON, Canada; BioRender; accessed [June 2026]; RRID: SCR_018361). Schematic illustration of the pathophysiological mechanisms underlying cardiovascular and renal toxicities associated with VEGF inhibition. Blockade of VEGF signaling reduces nitric oxide (NO) bioavailability, increases oxidative stress, impairs endothelial function, and promotes vasoconstriction, leading to hypertension. Altered renal sodium handling may further contribute to blood pressure elevation. Concurrent suppression of VEGF-dependent endothelial survival disrupts microvascular integrity, promotes capillary rarefaction, and compromises glomerular filtration barrier function, resulting in increased vascular permeability, proteinuria, and progressive renal dysfunction. Collectively, these alterations contribute to VEGF inhibitor-associated cardiorenal toxicity.

Collectively, disruption of physiological VEGF signaling provides a common mechanistic basis for the cardiovascular and renal adverse effects associated with VEGF-targeted therapy. The principal mechanisms underlying VEGF inhibitor-associated hypertension and renal injury are summarized in Figure 2. Among these complications, hypertension is the most frequent cardiovascular manifestation and reflects coordinated alterations in endothelial function, vasoactive signaling, and microvascular homeostasis, as discussed below.

### 7.1. VEGF Inhibition Causes Cardiovascular Toxicity

Hypertension is the most common dose-limiting cardiovascular adverse effect associated with VEGF-targeted therapies and represents a direct consequence of VEGF pathway suppression [2,6,24,34,36]. Under physiological conditions, VEGF signaling activates endothelial nitric oxide synthase (eNOS) through PI3K-AKT- and PLCγ-dependent pathways, thereby increasing NO production and maintaining vascular relaxation and endothelial homeostasis [6,34,41,42,61,62]. Accordingly, inhibition of VEGF signaling reduces NO bioavailability, resulting in endothelial dysfunction, increased vascular tone, and systemic hypertension [2,24,34,36].

Beyond impaired NO signaling, VEGF inhibition compromises endothelial survival and adaptive vascular responses, promoting oxidative stress, vascular stiffness, and progressive endothelial dysfunction [24,34,47,62]. VEGF is also required for maintenance of the microvasculature; therefore, prolonged pathway inhibition can promote capillary rarefaction, which increases systemic vascular resistance while reducing tissue perfusion and oxygen delivery [2,47]. These structural and functional vascular alterations can reinforce the effects of reduced NO bioavailability and contribute to the development and persistence of hypertension during VEGF-targeted therapy.

Dysregulation of vasoactive mediators provides an additional mechanism linking VEGF inhibition to cardiovascular toxicity. Reduced NO signaling is frequently accompanied by enhanced endothelin-1 (ET-1) activity, shifting vascular homeostasis toward vasoconstriction, vascular remodeling, and sustained blood-pressure elevation [93,106]. Renal mechanisms may further amplify this hypertensive response because VEGF inhibition can impair renal microvascular function and alter sodium handling, thereby promoting sodium retention and salt-sensitive hypertension [94,95]. Collectively, reduced NO bioavailability, endothelial dysfunction, oxidative stress, enhanced ET-1 signaling, microvascular rarefaction, and altered renal sodium handling form an interconnected mechanistic network underlying VEGF inhibitor-associated hypertension [47,93,94,95,96].

These cardiovascular effects extend beyond blood-pressure elevation. Persistent endothelial dysfunction increased vascular resistance, impaired microvascular perfusion, and vascular remodeling can contribute to broader cardiovascular complications associated with VEGF pathway inhibition [24,34,47,62]. Importantly, the kidney is not only involved in the hypertensive response but is itself a major target of VEGF pathway inhibition. The following section examines the glomerular, endothelial, and microvascular mechanisms underlying VEGF inhibitor-associated nephrotoxicity.

### 7.2. VEGF Inhibition Causes Renal Toxicity

The kidney is particularly susceptible to VEGF pathway inhibition because VEGF is essential for maintaining the structural and functional integrity of the glomerular filtration barrier. Within the glomerulus, VEGF produced by podocytes acts on adjacent glomerular endothelial cells to preserve endothelial fenestrations, capillary integrity, and normal filtration function [38,47,93]. Disruption of this podocyte–endothelial signaling axis following VEGFR2 inhibition promotes endothelial injury, loss of fenestrations, increased glomerular permeability, and proteinuria [93,94,95,96].

Proteinuria is a major manifestation of VEGF inhibitor-associated nephrotoxicity and, with sustained disruption of VEGF signaling, may be accompanied by progressive glomerular injury, glomerulosclerosis, renal fibrosis, and chronic kidney dysfunction [93,94,95]. These effects underscore the importance of VEGF not only in angiogenesis but also in maintaining the mature glomerular microvasculature and filtration barrier. ET-1 may further amplify renal injury. Enhanced ET-1 activity during VEGF pathway inhibition can promote renal vasoconstriction, glomerular hypertension, inflammation, oxidative stress, extracellular matrix accumulation, and fibrosis through endothelin receptor-dependent mechanisms [95,96,97,98,99,100]. Consistent with increased intrarenal endothelin activity, elevated urinary ET-1 excretion has been reported following VEGF pathway inhibition [95,98,99,100].

Renal microvascular rarefaction represents an additional mechanism linking VEGF inhibition to progressive kidney injury. VEGF normally supports endothelial survival, renal capillary density, and tissue oxygen delivery [100,101,102]. Accordingly, suppression of VEGF signaling can reduce microvascular density, impair renal perfusion and oxygen delivery, and promote tissue hypoxia [100,101,102,103,104,105]. The renal medulla is particularly vulnerable to disturbances in oxygen availability because it normally functions under relatively low oxygen tension. Further impairment of oxygen delivery can activate hypoxia-responsive mechanisms that promote tubular injury, inflammation, and interstitial fibrosis [3,18,102]. This process may establish a self-reinforcing cycle in which microvascular loss aggravates tissue hypoxia, hypoxia promotes fibrotic remodeling, and progressive fibrosis further compromises renal perfusion and function.

At the molecular level, suppression of VEGFR2 downstream signaling provides an important mechanistic link between VEGF inhibition and renal endothelial injury. Reduced PI3K-AKT activity compromises endothelial survival and NO-dependent vascular homeostasis, whereas inhibition of MAPK-ERK and PLCγ signaling further impairs endothelial integrity, vascular adaptation, and microvascular repair [18,61,103]. Collectively, these alterations promote endothelial dysfunction, glomerular barrier injury, microvascular rarefaction, tissue hypoxia, inflammation, and fibrogenesis, ultimately contributing to progressive renal impairment.

Collectively, disruption of podocyte–endothelial VEGF signaling, enhanced ET-1 activity, impaired VEGFR2-dependent endothelial survival, microvascular rarefaction, and tissue hypoxia provide an interconnected mechanistic framework for VEGF inhibitor-associated proteinuria and progressive kidney injury [47,93,94,95,96,97,98,99,100,101,102,103,104,105,106]. However, these renal abnormalities do not develop independently of the cardiovascular effects described above. Rather, VEGF inhibition simultaneously perturbs vascular and renal homeostasis, creating bidirectional interactions through which hypertension and systemic vascular dysfunction can aggravate renal injury, while renal microvascular dysfunction and altered sodium handling can further exacerbate cardiovascular stress.

### 7.3. Cardiorenal Interactions During VEGF Inhibition

Cardiovascular and renal toxicities associated with VEGF pathway inhibition are therefore mechanistically interconnected rather than independent adverse effects. The kidney contributes critically to long-term blood-pressure regulation, whereas systemic hypertension and vascular dysfunction can, in turn, exacerbate renal microvascular and glomerular injury. During VEGF inhibition, reduced NO bioavailability, enhanced ET-1 activity, endothelial dysfunction, and microvascular rarefaction increase systemic vascular resistance and promote hypertension [47,93,94,95,106]. Simultaneously, disruption of renal VEGF signaling impairs glomerular endothelial integrity and renal microvascular function, contributing to proteinuria, altered sodium handling, and progressive renal injury [93,94,95,96].

These cardiovascular and renal disturbances can reinforce one another through a bidirectional pathophysiological cycle. VEGF inhibition-associated impairment of renal microvascular function and sodium handling can promote sodium retention and salt-sensitive hypertension [94,97]. Conversely, sustained elevations in systemic blood pressure can increase hemodynamic stress on an already compromised renal microvasculature and glomerular filtration barrier. Enhanced ET-1 signaling provides an additional mechanistic link between the two organ systems because increased endothelin activity promotes systemic and renal vasoconstriction while also contributing to glomerular hypertension, oxidative stress, inflammation, extracellular matrix accumulation, and fibrosis [93,96,97,98,99,100,106].

Microvascular rarefaction and tissue hypoxia may further amplify this cardiorenal interaction. VEGF signaling is required for endothelial survival and maintenance of microvascular density; consequently, VEGF inhibition can impair tissue perfusion and oxygen delivery [100,101,102,103,104,105]. In the kidney, this effect is particularly relevant because further reductions in oxygen availability can activate hypoxia-responsive pathways that promote tubular injury, inflammation, and interstitial fibrosis [3,18,102]. Progressive renal injury may subsequently impair mechanisms involved in volume and blood-pressure regulation, thereby further increasing cardiovascular stress. Thus, vascular dysfunction, hypertension, renal microvascular injury, and tissue hypoxia can become mutually reinforcing components of VEGF inhibitor-associated cardiorenal toxicity.

At the molecular level, these interactions share a common foundation in suppression of VEGFR2-dependent endothelial signaling. Reduced PI3K-AKT activity impairs endothelial survival and NO-dependent vascular homeostasis, whereas inhibition of MAPK-ERK and PLCγ-dependent signaling compromises vascular adaptation and microvascular repair [18,61,103]. Together with enhanced ET-1 activity, these signaling disturbances provide a mechanistic connection between systemic vascular dysfunction and progressive kidney injury (Table 2).

Collectively, VEGF inhibitor-associated cardiovascular and renal toxicities represent an integrated cardiorenal response driven by disruption of endothelial homeostasis, altered vasoactive signaling, microvascular dysfunction, hypertension, and renal injury. Recognition of this interdependence has important clinical implications because changes in blood pressure, renal function, and proteinuria may reflect overlapping components of the same pathophysiological process rather than isolated complications. Accordingly, strategies to reduce VEGF-TKI-associated toxicity require coordinated cardiovascular and renal risk assessment, early monitoring, and mechanism-informed interventions that preserve organ homeostasis without unnecessarily compromising anticancer therapy. These considerations provide the basis for the toxicity-mitigation strategies discussed in Section 8.

## 8. Strategies to Mitigate VEGF-TKI-Induced Cardiovascular and Renal Toxicities

VEGFR-TKI-associated cardiovascular and renal toxicities reflect disruption of VEGF-dependent mechanisms that maintain endothelial integrity, vascular homeostasis, and renal function [6,24,34,36]. Management therefore requires a multidisciplinary approach that balances anticancer efficacy with cardiovascular and kidney safety [15,102,103,104]. The following subsections distinguish established clinical management from emerging mechanism-based strategies. Table 3 provides complementary context by summarizing established VEGF/VEGFR-targeted anticancer therapies, clinically evaluated combination approaches, and emerging or investigational strategies, with their current evidence level and principal limitations.

### 8.1. Proactive Monitoring and Risk Assessment

Comprehensive pretreatment assessment remains essential for identifying patients at increased risk for VEGF-TKI-associated complications [18,102,103]. Important considerations include pre-existing hypertension, chronic kidney disease, diabetes mellitus, cardiovascular disease, obesity, smoking history, and prior exposure to cardiotoxic therapies [18,102,103]. Baseline evaluation should include blood pressure measurement, serum creatinine, estimated glomerular filtration rate (eGFR), urinary protein assessment, and electrolyte monitoring. In selected high-risk patients, electrocardiography, echocardiography, and cardiac biomarker assessment may provide valuable baseline information [3,18,102,103]. Because treatment-related hypertension, proteinuria, and renal dysfunction often develop during the first months of therapy, continued surveillance throughout treatment is essential. Regular monitoring of blood pressure, renal function, and urinary protein excretion facilitates early detection of toxicity and timely intervention before progression to severe cardiovascular or renal complications [18,102]. Future risk-prediction models incorporating clinical variables and molecular biomarkers may further improve individualized monitoring strategies and toxicity prevention [3,18,103].

### 8.2. Management of VEGF-TKI-Induced Hypertension

Hypertension remains the most common cardiovascular toxicity associated with VEGF-TKIs and requires prompt treatment to reduce the risk of cardiovascular and renal complications [60,61,93,94,106]. Blood pressure should be assessed before treatment initiation and monitored regularly, particularly during early treatment cycles when elevations are most frequently observed [3,18,102]. Angiotensin-converting enzyme inhibitors (ACEIs) and angiotensin receptor blockers (ARBs) are particularly attractive because they provide both antihypertensive and renoprotective effects while reducing proteinuria and limiting progression of renal injury [60,61,105]. Calcium channel blockers such as amlodipine represent effective alternatives for patients without significant proteinuria [2,102,128]. Additional agents, including beta-blockers or selected diuretics, may be considered according to patient-specific comorbidities and treatment regimens [15,102]. Development of hypertension should not automatically necessitate discontinuation of VEGF-TKI therapy. In most cases, appropriate antihypertensive management permits continuation of treatment while minimizing cardiovascular risk. However, severe or refractory hypertension may require dose modification, temporary interruption, or specialized cardio-oncology consultation [105].

### 8.3. Emerging Mechanism-Based Therapeutic Strategies

Beyond established supportive care, several mechanism-based approaches are being investigated to preserve cardiovascular and renal function during VEGFR-TKI therapy. As summarized in Table 3, these strategies remain at different stages of development and should be distinguished carefully from clinically established management.

Among these investigational approaches, modulation of the epoxyeicosatrienoic acid (EET) pathway is of particular interest because of its endothelial, vascular, and renal protective effects. Strategies under investigation include soluble epoxide hydrolase (sEH) inhibition, dual sEH/COX-2 inhibition, and EET-based therapeutics; however, their efficacy for preventing VEGFR-TKI-associated cardiorenal toxicity has not yet been established clinically [31,69,124,126,127,128].

One approach involves inhibition of soluble epoxide hydrolase (sEH), the principal enzyme responsible for EET degradation [31,126]. In experimental animal models, sEH inhibition increases endogenous EET bioavailability, improves endothelial function, enhances nitric oxide signaling, reduces oxidative stress, preserves glomerular structure, and attenuates renal injury [31,126]. The dual soluble epoxide hydrolase/cyclooxygenase-2 inhibitor PTUPB has emerged as a promising preclinical candidate because it simultaneously preserves EET activity and suppresses inflammatory signaling pathways [31,69,126]. Preclinical studies suggest that PTUPB can attenuate cardiovascular and renal injury associated with VEGF-TKI exposure while maintaining antitumor activity in experimental models [31,69,126]. Nevertheless, these findings have not yet been validated in clinical trials involving cancer patients receiving VEGF-targeted therapies.

Additional EET-based approaches are also under active investigation. Kidney-targeted EET analogs such as EET-F01 have demonstrated renoprotective effects in experimental models by reducing renal inflammation, oxidative stress, and structural kidney injury [127,128]. Similarly, 8,9-EET analogs have shown protective effects against podocyte injury and glomerular dysfunction in in vitro and preclinical models of VEGF-TKI-associated nephrotoxicity [69]. Although these findings provide mechanistic proof-of-concept, evidence remains limited to experimental systems, and their efficacy and safety in humans remain to be determined through clinical investigation [69,124,126,127,128,134].

Beyond EET-based therapies, endothelin receptor antagonists represent another mechanism-based strategy under investigation. Given the established role of endothelin-1 in VEGF inhibitor-associated hypertension, endothelial dysfunction, and renal injury, selective ETA receptor blockade may offer therapeutic benefits by reducing vasoconstriction, inflammation, fibrosis, and proteinuria [88,94,95]. While endothelin receptor antagonists have demonstrated clinical efficacy in other cardiovascular and renal disorders, their role in preventing or treating VEGF-TKI-induced cardio-renal toxicity requires further clinical evaluation [97,98,106].

**Figure 3:**
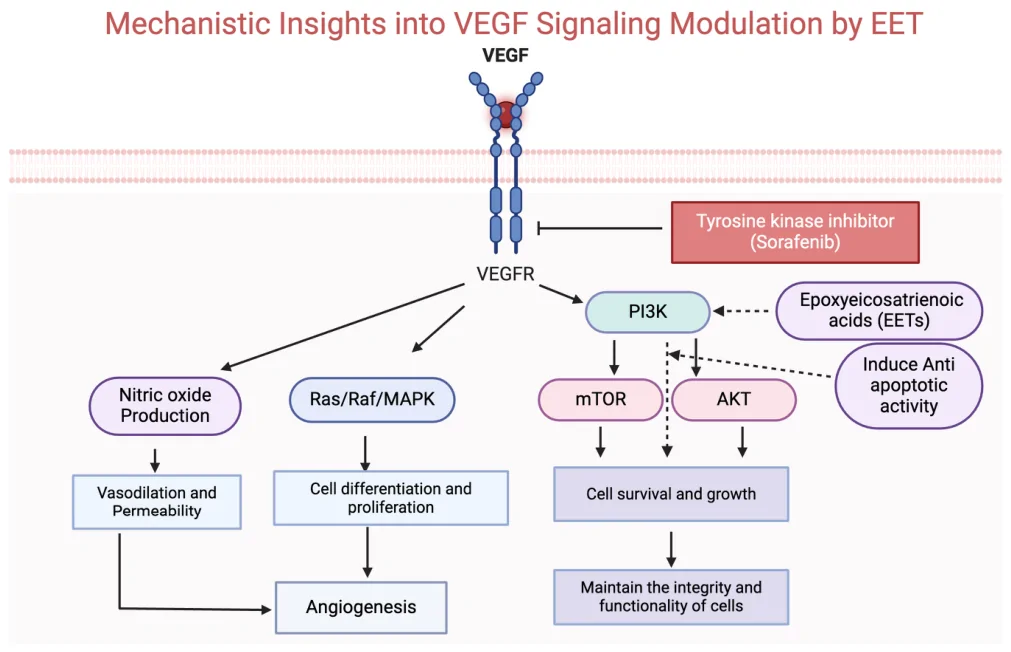
Proposed protective mechanisms of epoxyeicosatrienoic acids against VEGFR tyrosine kinase inhibitor-induced cardiovascular and renal toxicity. Graphical elements used in the figure were created using BioRender (BioRender, Toronto, ON, Canada; BioRender; accessed [June 2026]; RRID: SCR_018361). Schematic representation of the protective effects of epoxyeicosatrienoic acids (EETs) against VEGFR tyrosine kinase inhibitor (VEGFR-TKI)-induced cardiorenal injury. Activation of VEGF receptor signaling stimulates the PI3K/AKT, Ras/Raf/MEK/ERK (MAPK), and endothelial nitric oxide synthase (eNOS)/NO pathways, thereby promoting endothelial survival, vascular homeostasis, and tissue integrity. VEGFR-TKIs suppress these signaling cascades, resulting in endothelial dysfunction, oxidative stress, inflammation, hypertension, and renal injury. EETs enhance pro-survival signaling, preserve NO bioavailability, attenuate oxidative stress and inflammatory responses, and maintain endothelial and microvascular function. Through these complementary actions, EETs may mitigate VEGFR-TKI-associated cardiovascular and renal toxicities and may represent a potential therapeutic strategy for preventing VEGF inhibitor-induced cardiorenal injury.

In Figure 3, we schematically depict VEGFR blockade-induced suppression of NO, Ras/Raf/MAPK, and PI3K/AKT/mTOR signaling pathways and illustrate the proposed mechanisms by which EET-based therapies may restore endothelial survival signaling and mitigate VEGF-TKI-mediated cardio-renal injury.

### 8.4. Future Directions

Although VEGF-targeted therapies have transformed the management of many malignancies, their long-term use remains limited by cardiovascular and renal toxicities that can compromise treatment continuity and patient outcomes. Consequently, future research should prioritize strategies that preserve antitumor efficacy while minimizing treatment-related adverse effects [69,103,126,127,128,134]. Advances in biomarker-guided patient stratification, individualized risk prediction, precision dosing, and targeted drug-delivery platforms offer promising opportunities to enhance therapeutic selectivity and reduce unnecessary systemic VEGF suppression [103,104].

Natural compounds have also been investigated as experimental modulators of angiogenic and vascular signaling. Quercetin has been reported to influence VEGF/VEGFR, NF-κB, and PI3K/Akt pathways and to exert antioxidant and anti-inflammatory effects in experimental systems. Polydatin has likewise been studied for antioxidant, anti-inflammatory, and vasculoprotective properties. However, the evidence relevant to VEGF/VEGFR-targeted cancer therapy remains predominantly preclinical or review-level, and these compounds should not be considered established adjunctive treatments. Their potential utility requires rigorous characterization of bioavailability, pharmacokinetics, dosing, drug interactions, efficacy, and safety in appropriately designed clinical studies [130,131,132,133].

Particular emphasis should be placed on the prospective evaluation of EET-based therapies, soluble epoxide hydrolase inhibitors, endothelin receptor antagonists, and other vascular-protective interventions designed to maintain endothelial integrity, microvascular function, and renal health during anti-VEGF treatment [69,126,127,128,134]. As understanding of the molecular basis of VEGF-TKI toxicity continues to evolve, integration of advances from oncology, vascular biology, nephrology, and cardiovascular medicine will be essential for developing safer and more effective therapeutic strategies. Ultimately, the future of VEGF-targeted therapy will depend on achieving a more precise balance between sustained suppression of pathological angiogenesis and preservation of physiological vascular function.

### 8.5. Overall Summary and Future Perspectives

VEGF/VEGFR signaling integrates angiogenic and lymphangiogenic control with endothelial and organ homeostasis, making the pathway both a major anticancer target and a source of predictable on-target toxicity. Current VEGF-targeted agents provide important clinical benefit, but adaptive resistance and cardiovascular and renal adverse effects remain substantial limitations [24,47,57,61,62,64,65,66,67,68,69,73,75,90,93,94,95,96,97,98,99,100,101,102,103,104,106]. Established management therefore centers on risk assessment, surveillance, and clinically appropriate control of hypertension and renal complications, whereas EET-based therapies, soluble epoxide hydrolase inhibitors, PTUPB, endothelin-directed strategies, and natural compounds should presently be regarded as emerging or investigational approaches in this toxicity setting [69,97,106,124,126,127,128,130,131,132,133,134]. Future progress will depend on biomarker-guided treatment selection, rational combinations, and prospective clinical validation of protective strategies that preserve physiological vascular function without compromising antitumor efficacy [32,68,69,84,103].

## Figures and Tables

**Table 1:** Overview of vascular endothelial growth factor (VEGF) family ligands and their cognate receptors, major signaling pathways, and biological functions. The table summarizes the principal VEGF-VEGFR interactions, predominant signaling pathways, and major biological effects.

*Ligand*	*Receptor*	*Primary Function*	*Major Signaling Pathways*	*Key Biological Effects*	*References*
***VEGF-A***	VEGFR-1 (Flt-1)	Modulates VEGF availability and inflammatory signaling	Limited direct signaling	Angiogenesis regulation; monocyte/macrophage recruitment	[15,21,22,23]
***VEGF-A***	VEGFR-2 (KDR/Flk-1)	Principal angiogenic receptor	PI3K/Akt; PLCγ/PKC/MAPK	Endothelial proliferation, migration, survival, permeability, and angiogenesis	[21,24,25,26,27,28,29,30,31,32]
***VEGF-B***	VEGFR-1 (Flt-1)	Maintains vascular homeostasis	PI3K/Akt	Endothelial survival, vascular integrity, neuroprotection	[3,19,33]
***VEGF-C***	VEGFR-2 (KDR/Flk-1)	Angiogenic signaling	PI3K/Akt; MAPK/ERK	Angiogenesis and vascular remodeling	[7,19,34,35]
***VEGF-C***	VEGFR-3 (Flt-4)	Lymphatic endothelial activation	PI3K/Akt; ERK	Lymphangiogenesis and lymphatic maintenance	[19,20,25,35,36,37]
***VEGF-D***	VEGFR-2 (KDR/Flk-1)	Angiogenic signaling	PI3K/Akt; MAPK/ERK	Angiogenesis and vascular remodeling	[3,19,21,33]
***VEGF-D***	VEGFR-3 (Flt-4)	Lymphatic vessel growth	PI3K/Akt; ERK	Lymphangiogenesis and lymphatic function	[19,20,25,36,38]
***PlGF***	VEGFR-1 (Flt-1)	Pathological angiogenesis and inflammation	VEGFR-1-associated signaling	Tumor angiogenesis, ischemic and inflammatory responses	[15,21,22,23]

Abbreviations: Akt, protein kinase B; ERK, extracellular signal-regulated kinase; Flt-1, fms-like tyrosine kinase 1; Flt-4, fms-like tyrosine kinase 4; KDR, kinase insert domain receptor; MAPK, mitogen-activated protein kinase; PI3K, phosphoinositide 3-kinase; PKC, protein kinase C; PLCγ, phospholipase C gamma; PlGF, placental growth factor; VEGF, vascular endothelial growth factor; VEGFR, vascular endothelial growth factor receptor.

**Table 2:** Representative VEGFR Tyrosine Kinase Inhibitors in Cancer Therapy: Clinical Indications, Pivotal Trials, Efficacy Outcomes, and Major Adverse Effects. This table summarizes representative clinical evidence for VEGFR-TKIs across selected malignancies, including treatment setting, key efficacy outcomes, and major adverse effects.

*EGFR-TKI*	*Representative Indication*	*Pivotal Trial/Setting*	*Main Efficacy Outcome*	*Major Adverse Effects*	*Refs.*
***Sunitinib***	Metastatic RCC	First-line vs. interferon alfa	Median PFS, 11 vs. 5 months; ORR, 31% vs. 6%	Hypertension, fatigue, hand-foot syndrome, cytopenias, proteinuria	[107,108,109,110]
***Sunitinib***	Pancreatic neuroendocrine tumor	Advanced progressive disease vs. placebo	Median PFS, 11.4 vs. 5.5 months; ORR, 9.3% vs. 0%	Hypertension, diarrhea, fatigue, cytopenias	[108,109,110,111]
***Sorafenib***	Advanced HCC	First-line vs. placebo (SHARP)	Median OS, 10.7 vs. 7.9 months	Hand-foot skin reaction, diarrhea, hypertension	[108,109,110,112,113,114,115]
***Pazopanib***	Advanced/metastatic RCC	Randomized phase III vs. placebo	Median PFS, 9.2 vs. 4.2 months	Hypertension, diarrhea, hepatotoxicity, hair-color change	[108,109,110,114]
***Axitinib***	Metastatic RCC	Second-line vs. sorafenib (AXIS)	Median PFS, 6.7 vs. 4.7 months	Hypertension, diarrhea, fatigue, dysphonia	[108,109,110,116]
***Lenvatinib***	Radioiodine-refractory differentiated thyroid cancer	SELECT, vs. placebo	Median PFS, 18.3 vs. 3.6 months; ORR, 64.8% vs. 1.5%	Hypertension, proteinuria, diarrhea, fatigue	[108,109,110,117,118]
***Regorafenib***	Previously treated metastatic colorectal cancer	CORRECT, vs. placebo	Median OS, 6.4 vs. 5.0 months	Hand-foot skin reaction, hypertension, fatigue, diarrhea	[108,109,110,119,120,121]
***Cabozantinib***	Advanced RCC	METEOR, vs. everolimus	Median PFS, 7.4 vs. 3.8 months; ORR, 21% vs. 5%	Hypertension, diarrhea, fatigue, hand-foot syndrome	[108,109,110,122]

Abbreviations: AXIS, Axitinib Second-Line trial; HCC, hepatocellular carcinoma; ORR, objective response rate; OS, overall survival; PFS, progression-free survival; RCC, renal cell carcinoma; SELECT, Study of (E7080) Lenvatinib in Differentiated Cancer of the Thyroid; SHARP, Sorafenib HCC Assessment Randomized Protocol; TKI, tyrosine kinase inhibitor; VEGF, vascular endothelial growth factor; VEGFR, vascular endothelial growth factor receptor.

**Table 3:** VEGF/VEGFR-Targeted Anticancer Therapies and Emerging Therapeutic Strategies. This table summarizes established VEGF/VEGFR-targeted anticancer therapies, clinically evaluated combination strategies, and emerging or investigational approaches, highlighting therapeutic purpose, level of evidence, and major limitations.

*Strategy/Representative Agent(s)*	Primary Target/Mechanism	Therapeutic Purpose	Evidence/Clinical Status	Major Limitations/Key Safety Considerations	References
*VEGFR tyrosine kinase inhibitors (TKIs): sunitinib, sorafenib, pazopanib, axitinib, lenvatinib, regorafenib, cabozantinib*	VEGFR kinase inhibition; many agents also inhibit PDGFR, FGFR, KIT, RET, MET, and/or AXL	Established anticancer therapy that suppresses tumor angiogenesis across multiple solid tumors	Established/approved clinical anticancer therapy. Representative quantitative efficacy outcomes and major adverse effects are presented separately in Table 2	Hypertension, proteinuria, cardiovascular and renal toxicity, other agent-specific adverse effects, and intrinsic/acquired resistance	[17,60,61,107,108,109,110,123]
*Anti-VEGF monoclonal antibody: bevacizumab*	Neutralization of VEGF-A	Suppresses ligand-driven tumor angiogenesis; used alone or in disease-specific combination regimens	Established/approved clinical anticancer therapy in multiple cancer settings	Hypertension, proteinuria, thromboembolic/hemorrhagic complications, impaired wound healing, and resistance	[76,77,78,79,80,81,108,109,110,123]
*Oncologic VEGF ligand trap: ziv-aflibercept*	Soluble VEGFR1/VEGFR2-derived ligand trap that sequesters VEGF-A and PlGF	Systemic inhibition of VEGF-dependent angiogenic signaling in oncology	Established/approved oncologic VEGF-targeted therapy. Oncologic use should be distinguished from intravitreal aflibercept used for retinal disease	Systemic VEGF suppression may cause hypertension, proteinuria, and other vascular toxicities	[82,83,84,85]
*VEGF/VEGFR-targeted therapy + immune checkpoint inhibition*	Combined angiogenesis inhibition and immune-checkpoint blockade	Enhances antitumor activity by coupling vascular/microenvironmental modulation with restoration of antitumor immunity	Clinically established in selected malignancies/regimens; benefit is disease- and regimen-specific	Overlapping vascular, renal, and immune-mediated toxicities; patient selection and regimen-specific management are important	[80,81,86,87,88,89]
*VEGF + mTOR pathway inhibition: bevacizumab + temsirolimus/everolimus*	Concurrent VEGF ligand blockade and mTOR pathway inhibition	Targets complementary angiogenic and tumor-survival pathways and may address adaptive resistance	Clinically evaluated in phase I-III studies; efficacy is regimen- and treatment-setting-dependent, and bevacizumab + temsirolimus is not established for routine use	Substantial overlapping toxicity, frequent dose modification, and treatment discontinuation; benefit must be balanced against tolerability	[124,125]
*VEGFR2/VEGFR3 or VEGF-C/VEGF-D axis targeting*	Dual/interconnected inhibition of angiogenic and lymphangiogenic signaling	Investigational approach to limit tumor vascular remodeling, lymphangiogenesis, metastatic dissemination, and potential escape from VEGFR2-directed therapy	Experimental/investigational; supported mainly by mechanistic and preclinical evidence	Limited clinical validation; efficacy, target selectivity, and safety require further study	[26,27,56,57,58,59,60,61,62]
*Endothelin receptor antagonism*	Blockade of ET-1 signaling, particularly ETA-mediated vasoconstrictive and profibrotic pathways	Mechanism-based strategy to mitigate VEGF-inhibitor-associated hypertension, endothelial dysfunction, proteinuria, and renal injury	Emerging translational strategy. Endothelin receptor antagonists are clinically used in other disorders, but their role in VEGF-TKI-associated cardiorenal toxicity is not established	Requires prospective evaluation in VEGF-TKI-treated patients; potential class-specific safety and drug-interaction considerations	[97,98,106]
*Soluble epoxide hydrolase (sEH) inhibition: e.g., T-AUCB*	Increases endogenous EET bioavailability by inhibiting EET degradation	Experimental vascular and renal protection through preservation of endothelial function and attenuation of oxidative/inflammatory injury	Experimental/preclinical for VEGF-TKI-associated cardiorenal protection	No established clinical efficacy in patients receiving VEGF-TKIs	[31,126]
*Dual sEH/COX-2 inhibition: PTUPB*	Preserves EET signaling while inhibiting COX-2-dependent inflammatory pathways	Experimental strategy to reduce VEGF-TKI-associated cardiovascular and renal injury while maintaining antitumor activity in preclinical models	Preclinical candidate	Not validated in clinical trials for VEGF-TKI toxicity; human efficacy and safety remain unknown	[31,69,126]
*EET-based therapeutics: EET-F01 and 8,9-EET analogs*	EET-pathway-mediated cytoprotective, endothelial, and renal signaling	Experimental protection against renal, glomerular/podocyte, inflammatory, oxidative, and structural injury	In vitro/preclinical evidence; not clinically validated for VEGF-TKI toxicity	Human pharmacokinetics, dosing, efficacy, safety, and effects on antitumor therapy require clinical evaluation	[69,124,127,128]
*Natural compounds: quercetin, polydatin, and related phytochemicals*	Multi-pathway modulation, including VEGF/VEGFR-associated, inflammatory, antioxidant, and survival signaling	Potential antiangiogenic and/or vascular-protective adjunct concepts	Predominantly experimental/preclinical or review-level evidence; not established adjunctive therapy	Bioavailability, pharmacokinetics, dosing, drug interactions, efficacy, and safety require rigorous clinical validation	[129,130,131,132,133]

Note: Evidence ranges from established clinical use to experimental or preclinical investigation. Quantitative clinical outcomes for representative VEGFR-TKIs are summarized separately in Table 2. Combination strategies should be interpreted according to the specific regimen and treatment setting, as overlapping or enhanced toxicities may limit tolerability. Abbreviations: AXL, AXL receptor tyrosine kinase; COX-2, cyclooxygenase-2; EET, epoxyeicosatrienoic acid; ET-1, endothelin-1; ETA, endothelin type A receptor; FGFR, fibroblast growth factor receptor; KIT, KIT proto-oncogene receptor tyrosine kinase; MET, MET pro-to-oncogene receptor tyrosine kinase; mTOR, mechanistic target of rapamycin; PDGFR, platelet-derived growth factor receptor; PlGF, placental growth factor; RET, rearranged during transfection; sEH, soluble epoxide hydrolase; TKI, tyrosine kinase inhibitor; VEGF, vascular endothelial growth factor; VEGFR, vascular endothelial growth factor receptor.

## Data Availability

No new data were created or analyzed in this study. Data sharing is not applicable to this article.
